# Analyzing the microfoundations of human violence in the DRC - intrinsic and extrinsic rewards and the prediction of appetitive aggression

**DOI:** 10.1186/1752-1505-7-11

**Published:** 2013-05-17

**Authors:** Roos Haer, Lilli Banholzer, Thomas Elbert, Roland Weierstall

**Affiliations:** 1 Department of Politics and Public Administration, University of Konstanz, Universitaetstrasse 10, Konstanz, 78457, Germany; 2 Deutsches Institut für Entwicklungspolitik/German Development Institute, Tulpenfeld 6, Bonn, D-53113, Germany; 3 Department of Psychology, University of Konstanz, Universitaetstrasse 10, Konstanz, 78457, Germany

**Keywords:** Extrinsic rewards, Intrinsic rewards, Appetitive aggression, Democratic Republic of the Congo

## Abstract

**Background:**

Civil wars are characterized by intense forms of violence, such as torture, maiming and rape. Political scientists suggest that this form of political violence is fostered through the provision of particular intrinsic and extrinsic rewards to combatants. In the field of psychology, the perpetration of this kind of cruelty is observed to be positively linked to appetitive aggression. Over time, combatants start to enjoy the fights and even the perpetration of atrocities. In this study, we examine how receiving rewards (intrinsic versus extrinsic) influence the level of appetitive aggression exhibited by former combatants.

**Method:**

We surveyed 95 former combatants in the eastern provinces of the Democratic Republic of the Congo.

**Results:**

Linear regression analyses reveal that intrinsic as well as extrinsic rewards are linked to the former combatants’ Appetitive Aggression score. However, this relationship is partly determined by the way in which combatants are recruited: While abducted combatants seem to react more strongly to extrinsic rewards, the score of those that joined voluntarily is primarily determined by intrinsic rewards.

**Conclusions:**

We conclude that receiving rewards influence the level of appetitive aggression. However, which type of rewards (intrinsic versus extrinsic) is of most importance is determined by the way combatants are recruited.

## Background

Armed conflicts are characterized by severe cruelty. Combatants and civilians are not only killed in battles but in many instances the killing is accompanied by gruesome acts like torture, rape, or cutting ears, lips, breasts, and throats. This form of cruelty is a well-known phenomenon in conflict zones around the world [1]. It is mostly understood as a result of military weakness of the perpetrator [2], forms of military strategy to intimidate the enemy [3] measures to destroy the support base of the opposing party [4], and personal reasons such as rage [5]. Researchers, however, rarely take another perspective and examine these occurrences on the psychological level, by focusing on the underlying motivation of human aggression [6,7].

Aggressive behavior may be motivated by two quite different emotional conditions, opposite in valence: (1) The *reactive* aggression is elicited to fight off threat and danger, it serves to reduce aversive emotional arousal, such as anger or fear. (2) The appetitive form of aggression is rewarding in itself, it is perceived as appealing during the act of violence [8,9] and thus can sustain itself in a cycle of positive feedback. Previous authors have acknowledged that cruelty may be rooted in instrumental mechanisms of aggression [10] however, they have assumed that it is prompted by its anticipated benefits like in mating [11,12], status [13,14], or access to resources [15,16]. This seems as much of a misconception as assuming that sexual behavior would only be motivated by reproduction: Just as sexual acts can be enjoyable in themselves, so can acts of aggression. All too often, the biologically intrinsic joy of attacking and hunting is ignored by scholars when trying to explain the occurrence of violent acts although every culture has forms to live and enjoy appetitive aggression, such as boxing, gladiator fights or aggressive computer games.

Psychologists working in the field of civil war research have recently suggested that humans may develop high levels of appetitive aggression if they are exposed to violent settings in which the perpetration of violence is not only necessary to survive but perceived as rewarding and beneficial [17]. In samples with genocide perpetrators from Rwanda [18] and child soldiers from Uganda [19] appetitive aggression was related positively to committing violent acts and negatively correlated to developing the Posttraumatic Stress Disorder (PTSD). Notwithstanding, most of these studies, with perhaps the exception of Hecker et al. [20], have linked appetitive aggression to various psychological factors. However war, torture, and other severe cruel human right violations cannot only be understood on the individual level, but must also be considered on the societal and structural level, i.e., as a problem in politics or micro politics [21]. It is then also important to be aware of the political context in order to comprehend the meaning of organized violence for the individual and the community. This impairs not only the mental health and daily functioning on an individual level, but also compromises the reconstruction and development of society [21-24]. In order to fill this scientific niche, we will empirically investigate the influence of some identified micro political factors on the variation in appetitive aggression in a sample of former Congolese recruits from different armed groups in eastern provinces of the Democratic Republic of the Congo (DRC). This country is in a state of chaos and disintegration ever since its independence in 1960. The conflict is mainly about the control over natural resources (especially present in the eastern part) but it has also an ethnical component attached to it. It is even further complicated by the fact that some armed groups fighting the government troops, like the Rassemblement Congolais pour la Démocratie and the Forces Démocratiques de la Libération du Rwanda are supported by foreign states like Rwanda and Uganda. In the past decade, several new groups have emerged while others merge together. Officially a peace agreement was signed in 2003 and the United Nations was authorized to deploy peacekeepers. Although the presence of these peacekeepers has improved the situation in Congo considerable [25], the country is still faced with major challenges, among others, still active rebel groups raiding villages and committing severe human right abuses in combination with economic debt, inflation, unemployment, diseases, natural disasters and refugee flows.

In examining the influence of some identified micro political factors on the level of appetitive aggression in former Congolese combatants, we will focus on the role of rewards in motivating combatants for participation in armed groups – an issue that has evolved as a dominant theme in conflict research in the last decade [26-28]. This literature generally identifies two different types of rewards that can be granted to new recruits: The more evident ones are pecuniary or extrinsic rewards, which are those received benefits that are visible to others [29]. In industrial organizations, such rewards are distributed in the form of bonuses, raises or paid vacations. In armed groups, extrinsic rewards do not only take the form of money but also of food and/or recreational drugs such as alcohol or weapons. Besides extrinsic remunerations, people can also be motivated by non-pecuniary or intrinsic rewards, which are non-tangible incentives. While many types of “intrinsic” rewards refer to the individual such as self-determination, task involvement, curiosity, enjoyment and interest [30], the focus for military groups lies more on the collective than on the individual [31]. In armed units, intrinsic rewards come among others in the form of solidarity and attachment to the unit and fellow comrades [32]. Although the economic literature on the effect of rewards is reluctant to accept the power of these intrinsic soft rewards, especially social scientists examining the influence of social relationships and bonds in military groups, have acknowledged the power of these returns. Wong et al. [33] for example find that the strength of interpersonal bond between soldiers is a critical factor for combat motivation. Even though MacCoun et al. [34] dispute this finding that social bonds increase combat effectiveness, they also agree that “social bonds are profoundly important in soldiers’ lives”. The power of intrinsic rewards is then also important for other political spheres. Opp [35] for example finds that intrinsic rewards are major determinants for participation in protesting movements.

It is thereby acknowledged that rewards in general can foster specific types of behavior, including the perpetration of violent and aggressive acts, if this is the desired and promoted behavior [36]. While in civil society, violent behavior is often punished rather than promoted (and as such rewards work in the opposite directions), military groups need to endorse cruel actions. Humphreys and Weinstein [37] provide some first evidence that specific rewards may be related to military violence. On the basis of a large survey of ex-combatants in Sierra Leone, the authors argue that armed groups using more pecuniary rewards for attracting recruits are more engaged in higher levels of killings. In light of these results, we postulate in this study that the nature of rewards might have a significant influence on the level of appetitive aggression of individual combatants.

## Methods

### Participants

We interviewed 95 former members of different Congolese armed forces. These members were selected on the basis of a convenience sample. 94 participants completed the interview, only one indicated that he was too tired to finish the entire set of questions. Most of the participants had joined armed groups on a voluntarily basis (64%) (see also Table 1 for more descriptive statistics). The age of these former combatants ranged between 14 and 49 years (mean (*M)* = 21.47 years, standard deviation (*SD)* = 7.07 years). Only 5 participants were female. Of the 95 participants, 24 former combatants came from the province North Kivu and 61 from South Kivu. The other participants originated from other parts of the Congo or from other countries in eastern Africa. Unsurprisingly, more than half of the respondents had only a few years primary school and only 2 former combatants indicated to have a university degree. 57% had no job at the time of the interview.

**Table 1:** Descriptive statistics

**Variable**	**Recruitment method**	**Mean**	**St. Dev.**	**Min**	**Max**
AAS scores	Voluntarily	27.13	8.16	11	52
	Abducted	25.19	7.49	15	48
Closeness	Voluntarily	2.83	1.23	1	5
	Abducted	2.11	0.92	1	5
Money	Voluntarily	0.27	0.45	0	1
	Abducted	0.24	0.43	0	1
Duration (years)	Voluntarily	2.38	2.02	0	10
	Abducted	2.85	2.49	0	12
Combat actions	Voluntarily	147.11	425.38	0	2920
	Abducted	167.87	370.92	0	1460

In the sample we interviewed, several combatants indicated that they were more than once recruited into an armed organization (*M* = 1.67, *SD* = 0.80 groups, min = 1 and max = 4). Each of them was interviewed about his or her experience in every armed group in which he or she was involved.^a^ Of the participants, 32% interviewees were involved in one of the many different Mayi Mayi factions (a traditional community based Congolese militia group), 16% were involved in the Rassemblement Congolais pour la Démocratie (an armed group supported by Rwanda and Uganda), 10% were involved in the Les Forces Armées de la République Démocratique du Congo (the national army of the DRC), 8% were active in the L’Alliance des Forces Démocratiques pour la Libération du Congo (the armed group that overthrew Mobutu in 1997), 6% belonged to the Patriotes Resistants Congolais (a Congolese militia – closely related to the Mayi Mayi), another 6% were a member of Le Congrès National pour la Défense du Peuple (an armed group composed of mainly Tutsis fighting Congolose and Rwanda Hutu forces), 4% were active in the Forces Démocratiques de la Libération du Rwanda or the Interahamwe (an armed group primarily composed of abducted Congolese and former Rwandan Hutus that were active in planning and executing the genocide in Rwanda in 1994), and 3 other participants were unable to identify their armed group or were active in minor or unknown groups. See Prunier [38] for a more detailed description of the armed groups.

### Procedure

The semi-structured interview that forms the basis of this study was developed in collaboration with an interdisciplinary team consisting of political scientists and clinical psychologists with extensive experience in psychotraumatology from the University of Konstanz and the non-governmental organization (NGO) vivo international.^b^ The survey was conducted between March and May 2009 after pre-testing and receiving the permission of the ethics boards of the university. Most of the interviews took place in Bukavu, the capital of the Eastern DRC province South Kivu. Other interviews were held in Bunyakiri, a place in the same province where often battles between the major parties occur. The majority of the semi-structured interviews were conducted at three welcome centers, which provide shelter and elementary help for those combatants that are released, escaped or have been demobilized by different NGOs. All ex-combatants were offered participation and no one refused. Additionally, other former combatants were traced with the help of these centers and interviewed at their working place (20% of the sample). We interviewed all former combatants of two welcome centers, and some others from the third center.

In all settings, we assured as much confidentiality as possible: In the centers, the conversations were carried out in separate rooms with no attendance of any welcome center staff. At their working space, the interviews were carried out in discrete spaces to grant as much privacy as possible. Before the interview took place, participants were carefully informed about the purpose of this study and about their privacy and confidentiality rights. Informed consent was provided by a document that was signed by all people that were present during the interview (the participant, the researcher, and the translator), in which it was described that all the information given would be only used for scientific purposes and that the participant could stop the interview at any point of time. It was emphasized that the participant could always refuse to answer a question. Very importantly, we always explained carefully that participation would not result in any economic (or other) compensation or benefit.

Most of the interviews were carried out by two of the authors (RH and LB) while the third author (TE) carried out the pre-testing together with two locally experienced female researchers (who in addition to English and French also spoke either Swahili or Kinyarwanda and thus were able to control the translation). Interviews were in English and translated in the spoken language of the participant (Swahili, Kinyarwanda, or Lingala) by locally trained female or male translators. Additional conversations were also held in French, in cases where the respondent spoke this language. Before the interviewers were conducted, the translators received a training in which the researchers explained the purpose of each question. Translation issues were discussed during the training in order to avoid confusing during the interviews.

### Measures

#### Appetitive aggression scale

We assessed a person’s propensity towards perpetrating aggressive acts using the Appetitive Aggression Scale (AAS; Weierstall and Elbert [9]), a semi-structured interview for the assessment of a person’s propensity towards violence that has been validated with over 1.600 ex-combatants (including Rwandan genocide perpetrators and Ugandan child soldiers). Not only has the scale provided a valid assessment of a participant’s propensity towards violence, it has also been proven to have good psychometric properties [19]. The scale consists of 15 items, which has a Cronbach’s Alpha of 0.85.^c^ Since its publication, the AAS has been used in several other studies [20,39]. For each item, a statement regarding the appetitive perception of aggressive behaviour and manhunt was given and the participant had to decide how much he or she agreed with the statement. We asked them for example, to what extent he or she agreed with the statement “I get sexually aroused for killing people” or “I like to listen to people telling me stories about how they have killed others”. Responses were coded on a five-point Likert scale ranging from 0 (“I totally disagree”) to 4 (“I totally agree”). For the analyses the sum score was calculated. The total AAS score from the participants ranges from 11 to 52 points. See Table 1 for more descriptive statistics. The score of those participants that voluntarily joined the armed group range also from 11 and 52 with an average of 27.13 and a standard deviation of 8.16. The scores of abductees were somewhat smaller (*M* = 25.19, *SD* = 7.49, min = 15 and max = 48).

#### Intrinsic rewards

Because of their less visible quality, intrinsic rewards are difficult to measure. Generally, combatants who value their membership in the armed group and for whom cohesion, unity and comradry is important, should feel more intrinsically motivated to fulfill their tasks within the group and at the same time feel rewarded if the group acknowledges their actions. Conversely, combatants who do not feel attached to the armed group will not be intrinsically motivated to follow orders. For them, economic compensation, physical threat, or pressure will be the most important motivators for engaging in combat and for the perpetration of acts of violence. As a measure for intrinsic rewards, we assessed whether the combatant agree or disagreed with the statement that he or she feels still *close* to his or her former comrades in the group. This measure ranged from 1 (“I strongly disagree”) to 5 (“I strongly agree”) (*M* = 2.55, *SD* = 1.17).

#### Extrinsic rewards

Economic compensations, for example, in the form of extra payments or bonuses are common extrinsic rewards to foster and recompense a desired behavior. In organizations, extrinsic rewards are understood as rewards “provided by the organization for the purpose of facilitating or motivating task performance” [29]. As a measure for extrinsic rewards, we assessed whether the combatant ever received *money* for participating in fighting. On the basis of this question, we constructed a dichotomous variable (*M* = 0.26, *SD* = 0.44). See Table 1 for more descriptive statistics of this measure.

#### Duration

As the *duration* spend in the armed group could be a potential predictor for the development of appetitive aggression, we assessed the duration in years. This rests on the assumption that long exposures to violent stricken environments impact the personal behavior as individuals adapt to the hostile surrounding. This measure ranged from 0 to 12 years (*M* = 2.55 years, *SD* = 2.20) (*M*_*abductees*_ = 2.85 years, *SD*_*abductees*_ = 2.49; *M*_*voluntarily*_ = 2.38 years, *SD*_*voluntarily*_ = 2.02).

#### Number of combat actions

In concurrence with a cycle of violence theory and our previous results [40], it is hypothesized that those participants who have a higher propensity towards violence also engage in more combat actions, while in a loop this also fosters appetitive aggression. To control for this possible effect, we assessed the *number of self-reported combat actions*, i.e. we asked the participants how often they were involved in fighting. If they could not remember the exact number, we asked them to estimate the amount of fighting events that they were involved in an average week. On the basis of these questions we made a simple additive index ranging from 0 to 2920 (*M* = 154, *SD* = 405) for all participants (*M*_*abductees*_ = 167.87, *SD*_*abductees*_ = 370.92; *M*_*voluntarily*_ = 147.11, *SD*_*voluntarily*_ = 425.38). It is important to realize that this measure is subjective in the sense that it describes more the participant’s feeling on how often he or she was involved in fighting rather than the real number.

## Results

We used linear regression analysis to explore the association between these micro political variables and the propensity of appetitive violence. The statistical modeling and regression analysis was carried out with Stata version 11 and R version 2.15.1. We used the Akaike Information Criteria (AIC) to estimate the best model fit [41,42]. For the AIC, all possible competing forced entry regressions models are ranked according to their AIC, with the one having the lowest AIC being the best fitting, as the loss of information based on this model is the smallest. As the Variance Inflammation Factor (VIF) did not exceed (1.48) in all models, multicollinearity can be neglected.

The selected models are presented in Table 2. They include all of the predictors: closeness as intrinsic rewards, money as extrinsic rewards, duration, the number of self-reported combat actions, and the age of joining voluntarily or of abduction. In the first model information of all participants (those that are abducted and those that joined voluntarily) was included. However, some of the former combatants did not answer all questions, and were removed from the analysis. This model reveals that closeness (coeff. of 2.25, *p* <.001) and money (coeff. of 4.29*, p* <.01) are strong predictors for the AAS score. There is also a strong positive relation between the number of self-reported combat actions and the AAS score. The effect of the amount of time spent in the armed group are both not statistical reliable.

**Table 2:** AAS score prediction

**Variable**	**Model (1) all combatants**	**Model (2) abductees**	**Model (3) voluntarily**
Constant	18.04*** (1.98)	22.15*** (6.401)	14.44*** (2.75)
Closeness	2.25*** (0.68)	−1.99 (2.518)	3.43*** (0.85)
Money	4.29** (1.78)	8.10*** (2.80)	0.83 (2.33)
Duration (years)	−0.13 (0.32)	0.17 (0.45)	0.25 (0.50)
Combat actions	0.01** (0.00)	0.01 (0.00)	0.01** (0.01)
N	78	29	49
R^2^	0.26	0.44	0.32
R_adj_^2^	0.22	0.34	0.26

Note: coefficients are standardized; standard errors in parentheses; *** *p*<.001, ** *p*<.01, * *p*<.05.

To further examine this result, we performed two additional linear regression analyses for two sub-groups of combatants: One for participants that were abducted (Model 2) and another one for participants that joined voluntarily (Model 3). We deemed these additional tests necessary because we found indications in previous research that although appetitive aggression is present at least to some degree in both the abducted and the non-abducted group, it is more substantial in the latter group. In the two models, one can see that in both sub-groups, intrinsic and extrinsic rewards play different roles. For abductees, those who received money as a reward during their time in the armed group exhibited a higher level of appetitive aggression (coeff. of 8.10, *p* <.05). For those participants that joined on a voluntarily basis, it is an especially intrinsic reward, as the closeness that the participant feels towards his or her fellow combatants, determines their AAS score (coeff. of 3.43, *p* <.001).

In order to draw conclusion and to test whether there is indeed a significant difference between the coefficients of those that joined on a voluntarily basis and those that were abducted, we calculated another model, with all predictors of the AAS score including two additional interactions: closeness * recruitment method and money * recruitment method. The results are presented in Table 3 (*R*_adj_^2^ = .29).

**Table 3:** **Regression of AAS score**, **predictors**, **and interaction effects**

**Variable**	**Β**	**Std. Err.**	***p***
Constant	25.07	4.02	***
Money	7.18	3.10	**
Closeness	−2.20	2.10	
Recruitment	−9.98	5.02	*
Closeness * Recruitment	5.50	2.25	**
Money * Recruitment	−6.43	3.80	*
Duration (years)	0.22	0.35	
Combat actions	0.01	0.00	**
N		78	
R^2^		0.36	
R_adj_^2^		0.29	

Note: coefficients are standardized; *** *p*<.001, ** *p*<.01, * *p*<.05.

As can be seen from the table, both included interaction effects are significant predictors for the AAS score. The AAS score of abductees is more related to the extrinsic reward of money rather than the scores of those that joined the armed groups voluntarily (the coefficient is positive and significant). At the same time, the AAS score of the latter group is correlated with the intrinsic reward of feeling close and bonding with their fellow comrades (the coefficient is negative, *p*<.01).

## Discussion

If we want to understand the roots of genocide and mass killing, and the cruel violent actions that take place during these episodes, we need to look at the interplay between the mechanisms described in the field of international relations (political science) and the proposed attraction to and appetite for cruelty as is described in the field of psychology, as societal problems often provide a starting point for the outbreak of group violence [43]. This study is one of the first to bridge this gap by examining the relationship between rewarding (and its nature) and the appetitive effects of violence in humans. In doing so, we have relied on theoretical arguments put forth by scholars in international relations. Empirically, we examined this linkage with a sample of former combatants in the DRC. The AAS [9] assesses the extent of appetitive experience with the perpetration of violence or the infliction of harm that is aimed at a victim so the perpetrator experiences violent-related enjoyment by the exposure to violence cues such as the screaming and bleeding of the victim. Rewards, in turn were assessed with the question of whether the former combatant received money and whether he or she felt close to his or her former comrades.

The results indicate a significant and robust relationship between rewards and appetitive aggression. Additionally, we found that this relationship differs by method of recruitment. There was a stronger relationship between extrinsic (money) rewards and the AAS score for former abductees than for those that joined the armed group on a voluntary basis. At the same time, the AAS score of the voluntary joiners was determined by intrinsic rewards.

It is highly likely that this difference stems from their initial motivation and social position. Humphrey and Weinstein [44] find in their study about the functioning of armed groups in Sierra Leone that voluntary recruits stem largely from politically alienated social groups while abductees come from those groups that endure the most economic difficulties. It is then not surprising that fighters, who were forced to fight against their will, are less likely to establish bonds with other combatants as they do not share common goals. Coming mostly from poor backgrounds, however, even abducted combatants seem to be receptive to economic compensation as this type of reward seems to establish positive feelings towards the designated task of killing and harming. Voluntary members, on the other hand joined for a cause and are more likely to share common goals or ideologies with their comrades. This of course leads to a natural cohesion and vicinity within the ranks of the armed group. Even though combatants can also join voluntarily for economic reasons, for them, social recognition proves to be an important reward for their actions. In light of these findings we observe that extrinsic as well as intrinsic rewards can foster positive feelings towards aggression – which, in the anarchy like environment of the DRC results in the perpetration of horrible acts of cruelty.

However, it is important to note that this study has focused on one kind of extrinsic reward and one kind of intrinsic reward, money and social group bonding. Further studies are required to investigate the relationship between other examples of rewards and the AAS score. Additionally, it should be noted that the observed relationships between rewards and appetitive aggression is one of correlational nature, i.e. appetitive aggression might also for example determine whether an abductee receive money or not. The analyses also show that the duration of time spent in the armed group has no effect on the AAS score of the former combatant. Hecker et al. found similar results [20]. The number of self-reported combat actions is in contrast to duration, positively related to appetitive aggression, which also coincided with previous research [18].

### Limitations

This study provides first evidence of how received rewards relate to the AAS score. Notwithstanding, an important limitation is that we collected the data retrospectively. However, the exact nature of this bias is unknown. Furthermore, it can be assumed that those combatants who have a high amount of appetitive aggression are still eagerly involved in man hunting activities and thus unlikely to return to civil society and show up in our convenience sample. Additionally, it might be likely that armed groups select the more aggressive members in the first place. Future research, should then not only investigate to which extent this result can be generalized to different populations, but whether the identified relationships hold in larger samples. In addition, it might be interesting to investigate if there are particular group influences. Currently, we are unable to test specific group influences due to the uneven distribution of former combatants across the armed groups.

## Conclusions

The present investigation indicates the importance of theoretically combining insights of conflict studies (political science) and psychology. In doing so, we examined the relationship between receiving rewards and the level of appetitive aggression in former combatants. Empirically, this study shows that extrinsic rewards play an important role in predicting former abductees’ level of appetitive aggression, while intrinsic rewards have more influence on the AAS score of those combatants that joined on a voluntary basis.

## Endnotes

^a^ Some former combatants were recruited more than once by an armed group. These particular combatants answered the set of questions for each time they were recruited and consequently were more often used in the analysis. This might result in a bias, since observations are no longer independent. Consequently, we clustered the standard errors per individual across every model to confirm the robustness of our models. No significant changes could be identified between the clustered and unclustered models.

^b^ See for more information: http://www.vivo.org.

^c^ See Weierstall and Elbert [19] for more detailed information on the construction of the AAS.

## Competing interests

The authors declare that they have no competing interests.

## Authors’ contributions

RH: principal investigator, data collection, data analysis and manuscript preparation. LB: data collection and data analysis and manuscript preparation. TE: study design, pre-testing, manuscript preparation and supervisor. RW: Manuscript preparation. All authors read and approved the final manuscript.
